# Genetic Variations of ALDH (rs671) Are Associated With the Persistence of HBV Infection Among the Chinese Han Population

**DOI:** 10.3389/fmed.2022.811639

**Published:** 2022-02-14

**Authors:** Dabao Shang, Peng Wang, Weiliang Tang, Ruidong Mo, Rongtao Lai, Jie Lu, Ziqiang Li, Xiaolin Wang, Wei Cai, Hui Wang, Gangde Zhao, Qing Xie, Xiaogang Xiang

**Affiliations:** ^1^Department of Infectious Diseases, Ruijin Hospital, Shanghai Jiaotong University School of Medicine, Shanghai, China; ^2^Translational Laboratory of Liver Diseases, Department of Infectious Diseases, Ruijin Hospital, Shanghai Jiaotong University School of Medicine, Shanghai, China; ^3^Department of Hepatobiliary Medicine, Eastern Hepatobiliary Surgery Hospital, Naval Medical University, Shanghai, China

**Keywords:** HBV, rs671, ALDH, polymorphism, association study

## Abstract

Alcohol dehydrogenase 1B (ADH1B) and aldehyde dehydrogenase 2 (ALDH2), members of the alcohol dehydrogenase family, have important roles in liver diseases. The roles of the polymorphisms of ADH1B rs1229984 and ALDH2 rs671 in hepatitis B virus (HBV) susceptibility and persistent infection were investigated in the present study. Total 1,034 patients with hepatitis B [99 acute hepatitis B (AHB), 521 chronic hepatitis B (CHB), 158 acute-on-chronic liver failure (ACLF), 159 liver cirrhosis (LC), and 97 hepatocellular carcinoma (HCC)] and 1,262 healthy controls (HCs) of the Chinese Han population were recruited, and single nucleotide polymorphisms (SNPs) of rs671 and rs1229984 were genotyped. Independent and joint roles of rs671 and rs1229984 in HBV infection were analyzed. The results showed that rs671 genotypes had a significantly different distribution among different subgroups. Compared with HCs, the frequency of rs671-AA genotype was higher in hepatitis B individuals, especially in the CHB group [adjusted OR (95%CI) = 1.899 (1.232–2.928), *p* = 0.003, in the co-dominant model], which showed a significant positive association. It was further confirmed that CHB individuals who carried ALDH2 rs671-AA genotype had a higher risk of persistent HBV infection and higher HBV-DNA quantitation compared with those with GG/GA genotype. In addition, the rs671-AA genotype might predict HCC incidence in patients with CHB. There were no different distributions of alleles or genotypes in rs671 mutant among AHB, ACLF, LC, or HCC groups compared with HCs. These data suggested the possible hazardous role of rs671-AA variant in HBV infection and persistence.

## Introduction

Chronic hepatitis B (CHB) remains an important global health challenge due to high morbidity (~240 million hepatitis B virus (HBV) surface antigen carriers) (1) and mortality (7,86,000 people die each year from related complications) (2). The prevalence of HBV infection is still very high at about 7.18% in China, though huge improvements have been achieved *via* universal vaccination programs and effective antiviral treatments (3). Patients with chronic HBV infection have about a 10-fold higher incidence of hepatocellular carcinoma (HCC) and liver-related mortality than those without HBV infection (4). HBV infection is still a critical public health burden worldwide due to limited curable therapeutic options for HBV-related HCC and liver cirrhosis/failure.

It has been reported that HBV can enhance its DNA replication through the autophagy pathway mediated by HBV × protein (5, 6). Adenosine monophosphate-activated protein kinase (AMPK) is a crucial energy sensor in macroautophagy/autophagy and can restrict HBV replication through the promotion of autophagic degradation (7). The persistent activation of autophagy in hepatocytes during HBV infection might influence the persistence of HBV infection (8). This indicates that certain genes in the human body may have an impact on HBV infection.

Alcohol dehydrogenase 1B (ADH1B) and aldehyde dehydrogenase 2 (ALDH2), members of the alcohol dehydrogenase family, are crucial enzymes for alcohol metabolism (9). Recently, multiple studies have suggested that ALDH2 is involved in the course of autophagy in a variety of liver diseases. ALDH2 may inhibit metastasis in HCC cells by regulating the AMPK signaling pathway (10) and also ameliorate chronic alcohol-induced hepatic steatosis and inflammation through up-regulation of the autophagy pathway (11). The ALDH2 rs671 (Glu504Lys) mutant, a common missense mutation in the ALDH2 gene (12), has been found to increase protein turnover and promote hepatocarcinogenesis *in vivo* (13). Moreover, individuals with the ALDH2 rs671-AA genotype exhibit severely decreased activity of the ALDH2 enzyme and an elevated level of gamma-glutamyl transpeptidase (GGT) in patients with non-alcoholic fatty liver disease (NAFLD) (14), indicating that the polymorphism of ALDH2 might have an important influence on liver diseases.

However, there have been no studies focused on the relationship between ALDH2 rs671 mutant and HBV infection to date, and the role of the ALDH2 rs671 polymorphism in the pathogenesis of HBV infection. Therefore, the present study investigated the association and clinical relevance of ALDH2 polymorphisms with respect to HBV susceptibility and persistence in the Chinese Han population.

## Methods

### Subjects

A total cohort of 1,034 patients with HBV infection in the Southeastern China region was recruited from June 2011 to December 2014 in the Department of Infectious Diseases, Ruijin Hospital, Shanghai Jiaotong University School of Medicine. The Ethnically and geographically matched 1,262 HCs for a routine checkup were recruited from the Center of Health Examination of Ruijin Hospital in the same period.

The diagnosis of CHB was established by seropositivity of hepatitis B surface antigen (HBsAg) over 6 months according to the Chinese guideline of prevention and treatment for CHB (2010 version) (15) and did not have any other type of liver diseases, such as chronic hepatitis C, hepatitis D, hepatitis E, drug-induced liver diseases, and alcoholic or autoimmune liver disease. All participants were identified as Han Chinese. The demographic information included gender, age, birthplace, and past and current residency. The clinic data were collected from clinical records and/or telephone interviews. The study is approved by the Ethics Committee of Shanghai Ruijin Hospital, School of Medicine, Shanghai Jiaotong University in accordance with the Helsinki Declaration. The characteristics of AHB, CHB, LC, HCC, and HC are presented in Table 1.

**Table 1:** Demographic and clinical features of the patients and healthy controls in the study.

**Characteristic**	**HC**	**Total**	**I**	**II**	**III**	**IV**	**V**	**P_1_**	**P_2_**
	**(*n* = 1,262)**	**Hepatitis B (*n* = 1,034)**	**AHB (*n* = 99)**	**CHB (*n* = 521)**	**HBV-ACLF (*n* = 158)**	**HBV-LC (*n* = 159)**	**HBV-HCC (*n* = 97)**		
**Mean age** ^†^	46.49 ± 13.82	45.24 ± 13.45	39.78 ± 12.05	44.16 ± 13.72	45.06 ± 11.39	50.78 ± 9.836	55.94 ± 10.69	0.3276	<0.0001
**Gender**^††^ (Male/female)	734 (58.16)/ 527 (41.74)	628 (60.74)/ 406 (39.26)	56 (56.57)/ 43 (43.43)	331 (63.53)/ 190 (36.47)	87 (55.06)/ 71 (44.94)	91 (57.23)/ 68 (42.77)	63 (64.95)/ 34 (35.05)	0.455	0.0254
**ALT** (IU/ml)^†^	22.05 ± 9.095	283.6 ± 448.3	706.9 ± 668.6	290.9 ± 397.3	320.5 ± 577.1	90.24 ± 156.0	60.92 ± 53.93	<0.0001	<0.0001
**AST** (IU/ml)^†^	19.39 ± 4.951	175.4 ± 240.0	300.2 ± 325.2	165.2 ± 224.3	255.1 ± 316.1	89.71 ± 108.7	104.7 ± 1111.3	<0.0001	<0.0001
**Tbil** (umol/L)^†^	15.23 ± 4.265	139.9 ± 194.7	107.1 ± 117.2	80.10 ± 133.0	386.3 ± 198.4	96.41 ± 172.1	138.7 ± 235.8	<0.0001	<0.0001
**GGT (**IU/ml**)**^†^	18.47 ± 11.05	91.54 ± 99.15	145.2 ± 109.8	93.27 ± 84.86	74.98 ± 83.93	60.22 ± 72.33	111.9 ± 170.4	<0.0001	<0.0001
**AFP** (ug/L)^†^	1.982 ± 1.683	505.6 ± 3032	20.45 ± 45.33	203.9 ± 967	119.7 ± 167.9	110.4 ± 347.4	2,392 ± 6,529	0.0067	<0.0001
**eAg** **+** (no, %)^††^	/	550 (53.20)	52 (52.53)	237 (45.49)	69 (43.67)	49 (30.82)	28 (28.87)	/	0.0049
**HBV-DNA** [Log_10_ (copies)/ml]	/	5.491 ± 1.732	4.639 ± 1.46	5.971 ± 1.676	5.129 ± 1.549	5.022 ± 1.743	4.532 ± 1.731	/	<0.0001
<10^3^††^^	/	300 (29.05)	33 (33.33)	104 (19.96)	38 (24.05)	69 (43.40)	57 (58.76)	/	/
10^3^-10^5^††^^	/	279 (26.99)	43 (43.44)	125 (23.99)	51 (32.28)	43 (27.04)	18 (18.56)	/	/
>10^5^††^^	/	455 (43.96)	23 (23.23)	292 (56.05)	69 (43.67)	47 (29.56)	22 (22.68)	/	/
**Genotype** ^††^									
GG	709 (56.23)	547 (53.06)	59 (59.60)	260 (49.90)	79 (50.0)	92 (57.86)	60 (61.86)	/	/
GA	496 (39.33)	417 (40.45)	36 (36.36)	222 (42.61)	71 (44.94)	56 (35.22)	32 (32.99)	/	/
AA	56 (4.45)	67 (6.5)	4 (4.04)	39 (7.49)	8 (5.06)	11 (6.92)	5 (5.15)	/	/

*HC, health control; AHB, acute hepatitis B; CHB, chronic hepatitis B without ACLF, LC, and HCC; LC, HBV-related liver cirrhosis; HCC, HBV-related hepatocellular carcinoma;*

† *Date presented as (mean ± SD);*

†† *Date presented as (n, %); P_1_, t-test for all of the patients with CHB compared to HC individuals; P_2_, One-way ANOVA test for all of hepatitis B groups (groups I–V); Hepatitis B group is the sum of groups I–V*.

### Single Nucleotide Polymorphism Selection

Single nucleotide polymorphisms were selected using HapMap Data Rel 27 Phase II+III, February 2009, on NCBI B36 assembly, dbSNP b126 of Han Chinese Beijing (http://hapmap.ncbi.nlm.nih.gov/) and Haploview software 4.2 (Mark Daly's Lab of Broad Institute, Cambridge, MA, USA). The criteria used for the SNP selection were population-frequency and multiple, high-profile or inconsistent submitters. The core criterion was determined based on the alteration of ADH1B and ALDH2 transcription, translation, or function. Two SNPs (rs1229984 and rs671) were finally selected for the evaluation.

### Genomic DNA Extraction

Genomic DNA was extracted from 5 ml venous blood, using the DNA Extraction Kit (Tiangen Biotech Co., Ltd., Beijing, China) according to the manufacturer's instructions. After the determination of genomic DNA concentration, the samples were stored at −80°C until genetic polymorphism analyses.

### Genotyping

Rs671 was identified in the region of the ALDH2 gene on chromosome 12 (location on 111803962). Rs1229984 was identified in the region of the ADH1B gene on chromosome 4 (location on 99318162). SNP ID numbers and sequence are available at http://www.ncbi.nlm.nih.gov/snp/ (Supplementary Table 1). The primers used for the corresponding SNP PCR amplification and SNaPshot extension reactions were designed using the Primer 5 software (Supplementary Table 1). SNPs were confirmed by multiplex SNaPshot technology as previously described (16) using an ABI fluorescence-based assay allelic discrimination method (Applied Biosystems, Bedford, MA, USA).

The PCR was performed as described previously (17). Briefly, in a total volume of 20 μl containing 1 × ExTaq 0.2 μl, 25 Mm MgCl_2_ 2 μl, 25 mM dNTP mix 2 μl, (TaKaRa Bio, Dalian, China), 2 μl genomic DNA, and 4 μl of each primer. The PCR product was purified by 1 U shrimp alkaline phosphatase (SAP) and 1 U Exonuclease I. The product was processed according to the ABI SNaPshot protocol. The extension was performed in a total volume of 10 μl containing 5 μl SNaPshot Multiplex Kit (ABI), 2 μl PCR product, 1 μl mixed extension primer, and 2 μl H_2_O. The samples were put through 28 cycles of denaturation at 96°C, annealing at 50°C, elongation at 60°C, and a final extension at 72°C. The extension product was purified by 1 U SAP. The SNP genotype was confirmed using an ABI3130 genetic analyzer. Genotypes were determined automatically using the Genemapper 4.0 software (Applied Biosystems).

### Statistical Analysis

The significance was determined using Student's *t*-test or *Z*-test in demographic and clinical data for two groups or continuous variables. The χ^2^ test or the Fisher exact tests (two-sided) were used to compare the categorical variables. The differences between groups were examined using the respective genetics models of codominant, dominant, recessive, and additive, as appropriate. Statistical significance was performed using the SPSS software version 18.0 (SPSS Inc., Chicago, IL, USA) and GraphPad Prism 5 (GraphPad Software Inc., La Jolla, CA, USA). Hardy–Weinberg disequilibrium, the odds ratio with a 95% CI, logistic regression adjusted for age and gender were calculated by PLINK (v.1.07, http://pngu.mgh.harvard.edu/purcell/plink/, 5 February 2015, date last accessed) (18).

## Results

### Demographic and Clinical Characteristics

**Figure 1:**
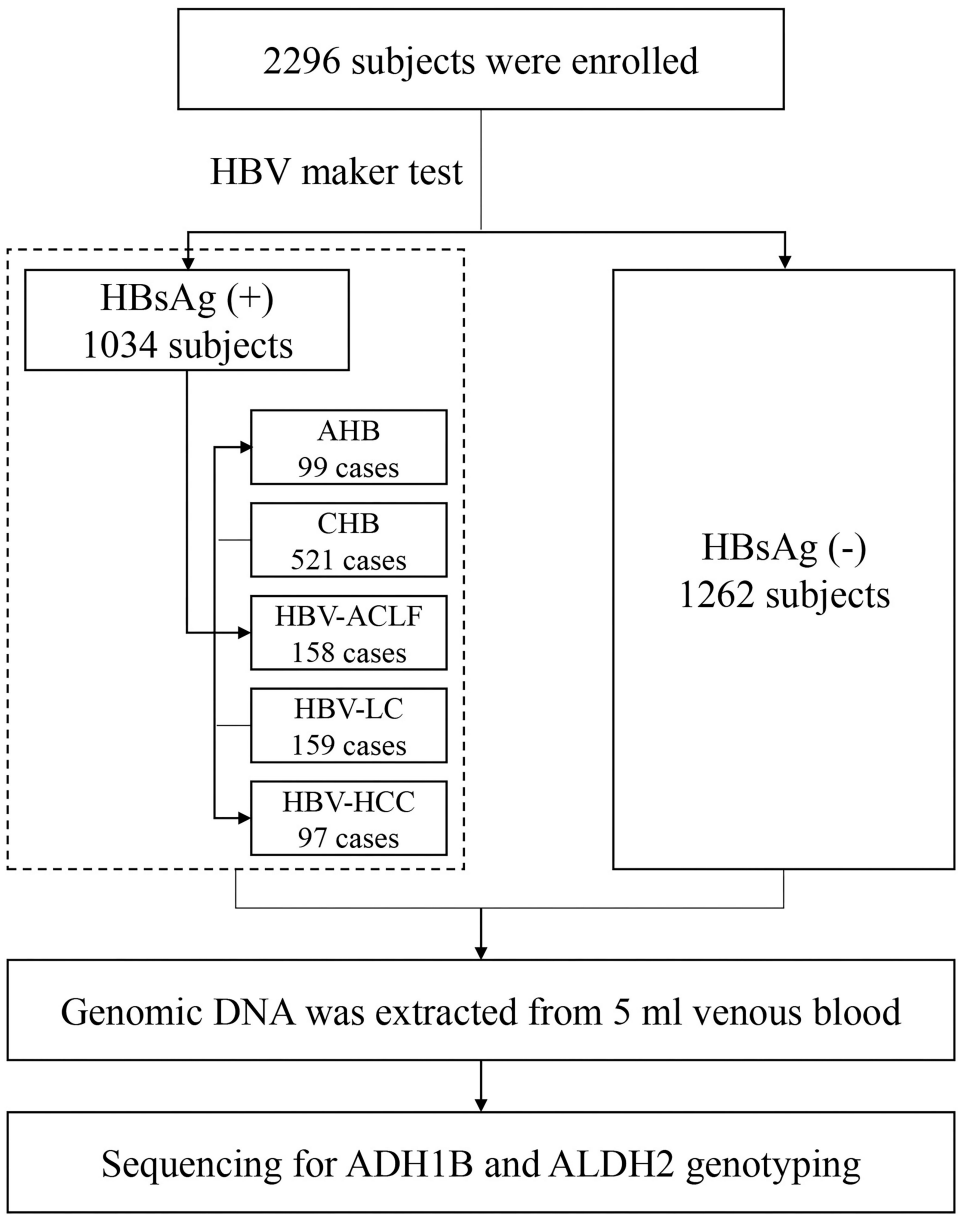
Flow chart of subjects included. HC, health control; AHB, acute hepatitis B; CHB, chronic hepatitis B without ACLF, LC, and HCC; LC, HBV-related liver cirrhosis; HCC, HBV-related hepatocellular carcinoma.

In total, 2,296 participants were recruited in the present study, including 1,034 patients infected with HBV [99 acute hepatitis B (AHB), 521 CHB without liver cirrhosis (LC) and HCC, 158 acute-on-chronic liver failure (ACLF), 159 HBV-associated liver cirrhosis (LC), and 97 HBV-associated HCC] and 1,262 HCs. The general demographic characteristics of the study population are shown in Table 1 and Figure 1. There were no significant differences in age and gender between the HBV group and the HCs. The mean age of the HBV group was similar to the HCs (45.24 ± 13.45 vs. 46.49 ± 13.82 years, *p* = 0.328). The levels of serum alanine aminotransferase (ALT), aspartate aminotransferase (AST), gamma-glutamyl transpeptidase (GGT), total bilirubin (TBIL), and alpha-fetoprotein (AFP) in the HBV group were significantly higher than that in HCs (all of *p* < 0.0001).

Similar to the clinical situation, there were different distributions of HBV-DNA quantity among the various subgroups (groups I, II, III, IV, and V). The quantity of HBV-DNA in patients with HCC was lower than that in the AHB, CHB, ACLF, or LC groups (*p* < 0.001). The positive percentage of hepatitis B e antigen (HBeAg) in the HCC group was also the lowest among all subgroups (*p* < 0.0049) (Table 1).

### Quality Assessment

In total, 2,849 variants of the 2 loci were successfully genotyped in the 2,296 samples. The rates of successful genotyping (call rate) were about 100% (Supplementary Table 2). Hardy–Weinberg disequilibrium was assessed using the Haploview 4.2 test. The genotype distributions of the 2 SNPs were consistent with the Hardy–Weinberg equilibrium in the HC and HBV groups (Supplementary Table 2). These results were suitable for further genetic analysis based on these quality control assessments.

### Positive Correlation Between ALDH2 rs671 AA/GA Mutants and HBV Infection

Minor allele frequency (MAF) between the patients with hepatitis B and HCs was compared in Table 2. The frequency of the A allele at rs671 in the HBV group was significantly higher than that in HCs [OR (95%CI) = 1.148 (1.004–1.312), *p* = 0.043]. The genetic models (codominant, dominant, recessive, and additive) were then applied to calculate genotype frequencies. The binary logistic regression was performed to analyze whether the variant on rs671 was independently associated with HBV infection. Age and gender covariates were included in the logistic regressions (19), which were previously reported to be significantly associated with HBV infection. In the codominant model, the frequency of the rs671-AA genotype in patients with hepatitis B accounted for a relatively high proportion [adjusted OR = 1.551 (1.069–2.25), *p* = 0.02], compared to HCs (Table 2). Similarly, the frequency of the GA + AA genotypes in patients with hepatitis B was significantly higher than that in HCs (43.77 vs. 46.94%, adjusted OR = 1.454, *p* = 0.046) in the recessive model (Table 2). Moreover, in the additive model, the frequency of the GA and AA genotypes at rs671 in patients with hepatitis B was significantly higher compared to HCs (adjusted OR was 1.226, *p* = 0.033) (Table 2).

**Table 2:** Association between rs671 and hepatitis B virus (HBV) infection in different hepatitis B groups.

**Model**	**HC**	**Hepatitis B**	**AOR**	***P***	**I**	**OR_**1**_**	**P_1_**	**II**	**OR_**2**_**	**P_2_**	**III**	**OR_**3**_**	**P_3_**	**IV**	**OR_**4**_**	**P_4_**	**V**	**OR_**5**_**	**P_5_**
**Alleles**																			
G	1914 (75.89)	1511 (73.29)	1		154 (77.78)	1		742 (71.21)	1		229 (74.47)	1		240 (75.47)	1		152 (77.55)	1	
A	608 (24.11)	551 (26.71)	**1.148 (1.004–1.312)**	**0.043**	44 (22.22)	0.899 (0.636–1.273)	0.55	300 (28.79)	**1.273 (1.082–1.497)**	**0.003**	87 (27.53)	1.196 (0.919–1.556)	0.182	78 (24.53)	1.023 (0.78–1.342)	0.867	42 (22.45)	0.742 (0.513–1.074)	0.112
**Codominant**																			
GG	709 (56.23)	547 (53.06)	1		59 (59.60)	1		260 (49.90)	1		79 (50)	1		92 (57.86)	1		60 (61.86)	1	
GA	496 (39.33)	417 (40.45)	1.09 (0.918–1.294)	0.326	36 (36.36)	0.872 (0.567–1.341)	0.533	222 (42.61)	1.221 (0.987–1.51)	0.066	71 (44.94)	1.285 (0.914–1.806)	0.148	56 (35.22)	0.87 (0.612–1.237)	0.437	32 (32.99)	0.648 (0.412–1.019)	0.041
AA	56 (4.44)	67 (6.49)	**1.551 (1.069–2.25)**	**0.020**	4 (4.04)	0.858 (0.301–2.45)	0.775	39 (7.49)	**1.899 (1.232–2.928)**	**0.003**	8 (5.06)	1.282 (0.589–2.787)	0.529	11 (6.92)	1.514 (0.765–2.994)	0.23	5 (5.15)	0.791 (0.278–2.253)	0.66
**Dominant**																			
GG+GA	1205 (95.56)	964 (93.51)	1		95 (95.96)	1		482 (92.51)	1		150 (94.94)	1		148 (93.08)	1		92 (94.85)	1	
AA	56 (4.44)	67 (6.49)	1.125 (0.951–1.329)	0.169	4 (4.04)	0.838 (0.547–1.285)	0.418	39 (7.49)	**1.272 (1.033–1.566)**	**0.023**	8 (5.06)	1.276 (0.915–1.78)	0.15	11 (6.92)	0.936 (0.666–1.315)	0.703	5 (5.15)	0.867 (0.562–1.336)	0.516
**Recessive**																			
GG	709 (56.23)	547 (53.06)	1		40 (40.40)	1		261 (50.10)	1		79 (50.00)	1		67 (42.14)	1		32 (32.99)	1	
AA+GA	552 (43.77)	484 (46.94)	**1.454 (1.006–2.1)**	**0.046**	59 (59.60)	0.855 (0.298–2.454)	0.771	260 (49.90)	**1.621 (1.056–2.488)**	**0.027**	79 (50.00)	1.072 (0.499–2.302)	0.858	92 (57.86)	1.61 (0.813–3.186)	0.171	65 (67.01)	1.306 (0.499–3.41)	0.586
Additive			**1.226 (1.016–1.479)**	**0.033**		0.862 (0.597–1.245)	0.429		**1.328 (1.066–1.654)**	**0.011**		1.095 (0.741–1.617)	0.65		1.234 (0.872–1.748)	0.236		1.103 (0.677–1.795)	0.694

*Data were presented as number (percentage) for every group. The differences in genotype frequencies between any two groups were analyzed using logistic regression models (codominant, recessive, dominant, and additive). Age and sex were included as covariates. ORs (adjusted odds ratio) were calculated and reported within the 95% CI. Groups I, II, III, IV, and V represented the AHB, CHB, ACLF, LC, and HCC groups, respectively. Hepatitis B group was all of the groups I, II, III, IV, and V. OR_1_, P_1_; OR_2_, P_2_; OR_3_, P_1_; OR_4_, P_4;_ and OR_5_, P_5_ were respectively calculated for groups I, II, III, IV, and V compared to the HC group. Significant p-values (< 0.05) are highlighted in bold*.

### Positive Association Between ALDH2 rs671-AA Mutant and HBV Persistence

When subgroup analysis was undertaken, different distributions of allele frequencies or genotypes in the rs671 mutant were only found between the HC group and CHB group (group II). When compared to HCs, a significantly higher frequency of the A allele at rs671 in the CHB group (group II) was found [OR (95%CI) = 1.273(1.082–1.497), *p* = 0.003]. Using the codominant genetic model, the AA genotype of rs671 significantly increased the risk of HBV infection in the CHB group (adjusted OR = 1.899, 95% CI = 1.232–2.928, *p* = 0.003), compared with the GG genotype. There was no significant difference in the rs671-GA genotype between the CHB group and HCs (adjusted OR = 1.221, 95% CI = 0.987–1.51, *p* = 0.066). The additional genetic model analysis also demonstrated that the rs671-AA mutant was positively associated with CHB, regardless of using the dominant model (GG vs. GA + AA, adjusted OR = 1.272, *p* = 0.023), recessive model (GG + GA vs. AA, adjusted OR = 1.621, *p* = 0.027) or additive model (adjusted OR = 1.328, *p* = 0.011) (Table 2). Additionally, compared with the rs671-GA genotype, the distribution of the rs671-AA genotype within the CHB group was also higher than that in HCs (adjust OR is 1.556, 95%CI: 1.004–2.412, *p* = 0.046), which suggested that the rs671-AA genotype was the dominant effect in patients with CHB (Table 2).

### Association Between ALDH2 rs671 Mutant and AHB, ACLF, LC, or HCC

For patients with AHB, ACLF, LC, and HCC, there were no differences in the rs671 allele frequencies, genotypes, or genetics models between patients and HCs, respectively. Binary logistic regression, adjusted for age and gender, also did not show any significant association between rs671 GA/AA and the risks of AHB, ACLF, LC, or HCC (Table 2).

When patients with CHB without LC/HCC (group II) were used as controls, there was a significantly decreased frequency of the rs671-AA genotype in patients with HCC [adjusted OR (95%CI) = 0.619 (0.385–0.994), *p* = 0.041 in the dominant model] (Table 3). These data suggest that the rs671-AA genotype might have a potential value for predicting a lower incidence of HCC in patients with CHB.

**Table 3:** Association between rs671 and hepatitis B virus (HBV) persistence among hepatitis B subgroups.

**Model**	**II**	**I**	**OR_**1**_**	**P_1_**	**III**	**OR_**2**_**	**P_2_**	**IV**	**OR_**3**_**	**P_3_**	**V**	**OR_**4**_**	**P_4_**
**Alleles**													
G	742 (71.21)	154 (77.78)	1		229 (74.47)	1		240 (75.47)	1		152 (77.55)	1	
A	300 (28.79)	44 (22.22)	0.707 (0.493–1.014)	0.058	87 (27.53)	0.939 (0.71–1.245)	0.664	78 (24.53)	0.804 (0.602–1.073)	0.138	42 (22.45)	**0.683 (0.473–0.987)**	**0.041**
**Codominant**													
GG	260 (49.90)	59 (59.60)	1		79 (50)	1		92 (57.86)	1		60 (61.86)	1	
GA	222 (42.61)	36 (36.36)	0.717 (0.455–1.123)	0.144	71 (44.94)	1.053 (0.729–1.52)	0.785	56 (35.22)	0.713 (0.489–1.04)	0.078	32 (32.99)	**0.625 (0.392–0.994)**	**0.044**
AA	39 (7.49)	4 (4.04)	0.452 (0.155–1.314)	0.136	8 (5.06)	0.675 (0.303–1.505)	0.334	11 (6.92)	0.797 (0.392–1.622)	0.531	5 (5.15)	0.556 (0.21–1.469)	0.231
**Dominant**													
GG+GA	482 (92.51)	95 (95.96)	1		150 (94.94)	1		148 (93.08)	1		92 (94.85)	1	
AA	39 (7.49)	4 (4.04)	0.653 (0.419–1.016)	0.058	8 (5.06)	0.977 (0.682–1.398)	0.897	11 (6.92)	0.748 (0.513–1.089)	0.129	5 (5.15)	**0.619 (0.385–0.994)**	**0.041**
**Recessive**													
AA+GA	261 (50.10)	40 (40.40)	1		79 (50.00)	1		67 (42.14)	1		32 (32.99)	1	
GG	260 (49.90)	59 (59.60)	0.485 (0.169–1.397)	0.18	79 (50.00)	0.692 (0.314–1.524)	0.361	92 (57.86)	1.104 (0.536–2.275)	0.789	65 (67.01)	1.119 (0.409–3.063)	0.827
Additive			0.647 (0.378–1.106)	0.111		0.836 (0.559–1.252)	0.386		0.98 (0.677–1.419)	0.916		0.949 (0.568–1.584)	0.841

*Data were presented as number (percentage) for every group. The differences in genotype frequencies between any two groups were analyzed using logistic regression models (codominant, recessive, dominant, and additive). Age and sex were included as covariates. ORs (adjusted odds ratio) were calculated and reported within the 95% CI. Groups I, II, III, IV, and V represented the AHB, CHB, ACLF, LC, and HCC groups, respectively. OR_1_, P_1_; OR_2_, P_2_; OR_3_, P_3_, and OR_4_, P_4_ were respectively calculated for Group I, III, IV, and V compared to the II group. Significant p-values (< 0.05) are highlighted in bold*.

### No Association Between ADH1B rs1229984 Mutant and HBV Infection

Genotyping of rs1229984 (His48Arg) of ADH1B and rs671 (Glu504Lys) of ADH1B were performed using 266 patients with CHB without LC/HCC and 287 HCs of the Chinese Han population (Table 4). The results are shown in Table 4 and Supplementary Table 3. The call rates for rs1229984 and rs671 were 100%. Those variants in the control and case-patient group were in accord with Hardy–Weinberg equilibrium (*p* > 0.05).

**Table 4:** CHB risk due to the combination of ADH1B and ALDH2 genotypes.

**rs1229984 (His48Arg)**	**rs671(Glu504Lys)**	**HC (*n* = 287)^††^**	**CHB (*n* = 266)^††^**	**OR (95%CI)**	***p*-value**
AA	GG	77	60	1	/
AA	GA	52	60	1.481 (0.896–2.446)	0.245
AA	AA	7	5	0.917 (0.277–3.033)	0.194
AG	GG	59	54	1.175 (0.712–1.937)	0.618
AG	GA	57	57	1.283 (0.779–2.113)	0.974
AG	AA	7	2	0.367 (0.073–1.830)	0.849
GG	GG	16	15	1.203 (0.551–2.628)	0.298
GG	GA	12	12	1.283 (0.538–3.059)	0.940
GG	AA	0	1	3.843 (0.154–96.09)	0.649
His+ (AA/AG)	Lys- (GG)	136	114	1	/
His- (GG)	Lys+ (AA/GA)	12	13	1.292 (0.567–2.944)	0.675
His- (GG)	Lys- (GG)	16	15	1.118 (0.53–2.361)	0.849
His+ (AA/AG)	Lys+ (AA/GA)	123	124	1.203 (0.923–1.294)	0.324

HC, health control; CHB, chronic hepatitis B; His, histidine; Lys, lysine; OR, odds ratio; CI, confidence interval. ^*^In the analysis of rs1229984 (His48Arg), “His+” and “His–” mean His carrier (His/His or His/Arg) and non-His carrier (Arg/Arg), respectively. In the analysis of rs671 (Glu504Lys), “Lys+” and “Lys–” mean Lys carrier (Lys/Lys or Lys/Glu) and non-Lys carrier (Glu/Glu), respectively. We investigated the combined effects of rs1229984 and rs671 on CHB as compared with “His +/Lys–”.

†† *Data were presented as number (percentage) for every group. The differences in genotype frequencies between any two groups were analyzed using logistic regression models. Age and sex were included as covariates. The P-values were calculated for CHB patients compared to HC individuals*.

The variant of ADH1B rs1229984 showed no association with CHB in allele frequencies analysis [*p* = 0.996, OR (95%CI) = 1.001 (0.771–1.299)], or genotype models analysis [adjusted *p* = 0.785, OR (95%CI) = 0.947 (0.662–1.353), in the co-dominant model] (Supplementary Table 3). The joint effects of the combined variants of ADH1B (rs1229984) and ALDH2 (rs671) on the HBV persistent infection were investigated. Based on the enzyme activity (20), the His carrier (His+) and non-His carrier (His–) models were selected for the association analysis between CHB and rs1229984 (His48Arg) of ADH1B. The His carrier is mainly represented as rs671-AA/GA genotypes and the non-His carrier is mainly represented as the rs671-GG genotype. Regarding the association analysis between CHB and rs671 (Glu504Lys) of ALDH2, we adopted the non-Lys carrier (Lys–) and the Lys carrier (Lys+) models as described previously (21). The non-Lys carrier or the Lys carrier respectively represented AA or GG/GA genotypes. There are no significant differences in the distribution of alleles and genotypes between HCs and CHBs, according to a subgroup analysis of His–/Lys+, His–/Lys–, His+/Lys+, and His+/Lys+, as shown in Table 4 (*p* = 0.675, 0.849, and 0.324, respectively). There were also no significantly combined effects in CHB of the variants of ADH1B (rs1229984) and ALDH2 (rs671).

### Positive Association Between ALDH2 rs671-AA Mutant and the HBV-DNA Quantitation

**Figure 2:**
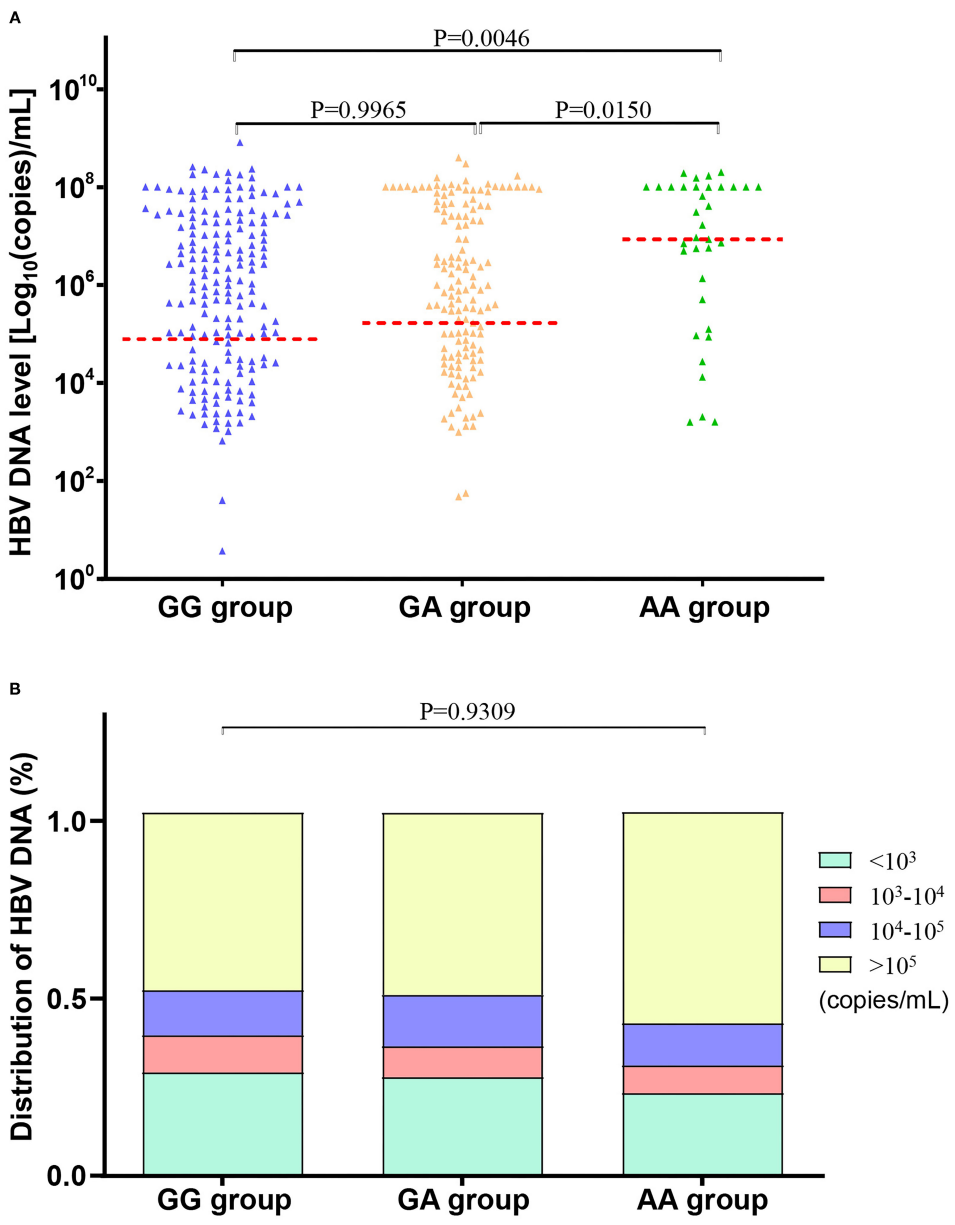
HBV-DNA levels were the highest in patients with chronic hepatitis B (CHB) with rs671-AA genotype. **(A)** Comparison of HBV-DNA mean levels among subjects with AA, AG, and GG genotypes at rs671 in the CHB group. HBV-DNA levels were analyzed by converting to Log_10_ (copies)/ml. Data were presented as (mean ± SD), unpaired *t*-test, and one-way ANOVA were used. **(B)** Comparison of HBV-DNA distribution among subjects with AA, AG, and GG genotypes at rs671 in the CHB group. Patients with CHB with rs671-AA genotype showed the highest proportion (58.97%) of HBV-DNA levels at more than 10^5^ copies/ml, compared to patients with rs671-GG (50.45%) or rs671-GA (49.23%). Data were presented as a percentage for each group. Fisher's exact test was used.

The CHB subjects with the rs671-AA genotype were found to have a significantly higher quantity of circulating HBV-DNA [6.745 ± 1.603 Log_10_ (copies)/ml] than those with rs671-GG [5.877 ± 1.651 Log_10_ (copies)/ml] or rs671-GA [5.980 ± 1.650 Log_10_ (copies)/ml] genotype (*p* = 0.0046), as shown in Table 5 and Figure 2A. There was a higher percentage of patients with CHB with a high quantitation of HBV-DNA (>10^5^ copies/ml) in the individuals with rs671-AA (58.97%), compared with patients with rs671-GG (50.45%) or rs671-GA (49.23%) (Table 5 and Figure 2B). It was demonstrated that the rs671-AA mutant was positively correlated with the quantitation of HBV-DNA.

**Table 5:** Comparison of clinical features levels between subjects with different genotypes at rs671 in the CHB (*n* = 521) group.

**Characteristic**	**GG (*n* = 260)**	**GA (*n* = 222)**	**AA (*n* = 39)**	*P* ^*^
**Mean age** ^†^	42.01 ± 13.88	43.81 ± 14.18	43.46 ± 13.90	0.169
**Gender**^††^ (Male/female)	157 (60.38)/ 103 (39.62)	148 (66.67)/ 74 (33.33)	28 (71.79)/ 11 (28.21)	0.115
**ALT** (IU/ml)^†^	282.7 ± 379.0	308.2 ± 424.3	385.7 ± 448.3	0.338
**AST** (IU/ml)^†^	162.3 ± 217.7	173.1 ± 237.2	213.1 ± 243.2	0.512
**Tbil** (umol/L)^†^	78.01 ± 127.5	87.61 ± 142.7	98.90 ± 150.3	0.974
**GGT (**IU/ml**)**^†^	94.34 ± 84.63	92.20 ± 82.72	91.91 ± 101.3	0.962
**AFP** (ug/L)^†^	210.2 ± 931.5	242.0 ± 1,144	86.04 ± 211.4	0.743
**eAg** **+** (no, %)^††^	171 (65.77)/ 89 (34.23)	145 (65.32)/ 77 (34.68)	29 (74.36)/ 10 (25.64)	0.161
**HBVDNA** [Log_10_ (copies)/ml]	5.877 ± 1.651	5.980 ± 1.650	6.745 ± 1.603	**0.0046**
<10^3^^††^	74 (28.46)	60 (27.03)	9 (23.08)	/
10^3^-10^4^††^^	26 (10.00)	19 (8.56)	3 (7.69)	/
10^4^-10^5^††^^	32 (12.31)	31 (13.96)	4 (10.26)	/
>10^5^^††^	128 (49.23)	112 (50.45)	23 (58.97)	**<0.0001**

† *Data presented as (mean ± SD)*.

†† *Data were presented as number (percentage) for every group*.

* *Difference in clinical features levels was tested between different genotypes (AA, AG, and GG) by one-way ANOVA test. Significant p-values (< 0.05) are highlighted in bold*.

As for age, gender, liver function (including ALT, AST, GGT, and TBIL), AFP, and rate of positive HBeAg, there were no significant differences among patients with CHB with GG, GA, or AA genotype (Table 5).

## Discussion

In the current study, the association between polymorphisms within the ADH1B/ALDH2 genes and HBV susceptibility was investigated in the Chinese Han population. It was the first investigation that focused on the relationship between the ALDH2 rs671 polymorphism and HBV susceptibility of individuals till now. It was also the first study that has demonstrated that individuals who carried ALDH2 rs671-AA genotype might have a higher risk of persistent HBV infection and higher HBV-DNA level compared to those with GG/GA genotype. Our results suggest the possible hazardous role of this variant during persistent HBV infection. Based on the fact that the rs671 (Glu504Lys) SNP has been shown to be a well-known dysfunctional SNP in a previous study (22), which has been confirmed in the present data, it is reasonable to conclude that the rs671-AA genotype is a potential hazardous HBV-associated functional SNP.

In addition, the rs671-AA variant might be a risk predicator for the incidence of HCC in patients with CHB, suggesting that patients with CHB with the rs671-AA genotype might have a lower risk of HCC incidence. However, there was no significant different distribution of allele or genotypes in the rs671 mutant among patients with AHB, ACLF, LC, or HCC, compared with HCs. The results further indicate that patients with CHB with persistent high HBV-DNA replication might be influenced by the rs671 polymorphism of ALDH2, whereas HBV-DNA replication in AHB, ACLF, LC, or HCC was lower than in CHB. It is worth initiating studies to reveal the underlying mechanism of the rs671 mutants and HBV replication.

The ALDH2 is a crucial enzyme in the hepatocyte, which takes part in alcohol metabolism. Alcohol is oxidized to acetaldehyde by ADH, and acetaldehyde is further metabolized to acetate by ALDH, which largely depends on ALDH2 (22). ALDH2 also plays important role in other liver diseases, including ameliorating chronic alcohol-induced hepatic steatosis and inflammation (11), inhibiting aggressive behavior of HCC (10), and increased risk for NAFLD with a mutation in ALDH2 (14).

The ALDH2 rs671 (Glu504Lys) is a common missense SNP, mainly in East Asians (40–50%), resulting in a Gly-to-Lys amino acid substitution in exon 12 (23). Individuals with the ALDH2 rs671-AA genotype exhibit severely decreased activity of the ALDH2 enzyme and have only 6.25% of the normal protein encoded by the ALDH2 rs671-GG variant, indicating the dominant effect of the ALDH2 A allele (24–26). Murine models with the rs671-AA mutant on ALDH2 could increase protein turnover and would promote murine hepatocarcinogenesis *in vivo* (13). It had been reported that the ALDH2 rs671-AA, which is associated with the GGT level, might potentially be a novel risk factor for NAFLD (14). In our study, we also found that individuals who carried the ALDH2 rs671-AA genotype had a higher risk of persistent HBV infection and higher HBV-DNA levels, compared to subjects with the rs671-GG genotype.

At first, we found that the distribution frequency of the A allele on ALDH2 rs671 was increased in patients with hepatitis B, especially in the CHB group, compared with HCs. However, no significant difference in the distribution of allele or genotype was found in rs671 mutants among patients with AHB, ACLF, LC, or HCC, compared with HCs, suggesting that the potential role of the rs671-AA variant is mainly related to the persistent HBV infection (CHB). We further found that the ALDH2 rs671-AA genotype was significantly increased in the CHB group compared with HCs, whereas the rs671-GA genotype was not significantly increased. The results demonstrated that the rs671-AA genotype might play a dominant effect on the HBV persistent infection. In addition, compared to the rs671-GA genotype, individuals with the rs671-AA genotype were significantly higher in the CHB group than that in HCs, which also suggested the dominant effect on CHB. We reasonably concluded that the rs671-AA genotype, not the rs671-GA genotype, might have an influence on the persistence of HBV infection.

Recently, cumulative evidence has revealed that ALDH2 plays an important role in liver diseases associated with the autophagy signal pathway (27, 28). ALDH2 could ameliorate chronic alcohol intake-induced hepatic steatosis and inflammation through the regulation of autophagy (11). Moreover, upregulating the expression of ALDH2 in HCC cells leads to the inhibition of tumor aggressive behavior *in vitro* and *in vivo*, largely exerted by modulating the activity of the ALDH2–acetaldehyde–redox–AMPK axis, which is an important autophagy pathway (10).

It has been reported that the enhancement of autophagy could increase HBV-DNA replication mediated by HBV × protein (5, 6), and the promotion of autophagic degradation by AMPK could restrict HBV replication (7). Meanwhile, HBV evaded antiviral immunity and permitted survival of virus-infected cells through triggering autophagy by the degradation of the TNFSF10/TRAIL response, which targets the TNFRSF10B/death receptor 5 (29). Moreover, the inhibition of ALDH2 activity could result in upregulated inflammatory molecules, including an increase of nuclear translocation of NF-κB and the enhancement of phosphorylation of NF-κB, p65, AP-1 c-Jun, Jun-N terminal kinase, and p38 MAPK (30). The persistent activation of autophagy and the inflammatory response in hepatocytes, which is mediated by ALDH2 during chronic HBV infection, might take part in the regulation of HBV infection and lead to persistent infection (8).

**Figure 3:**
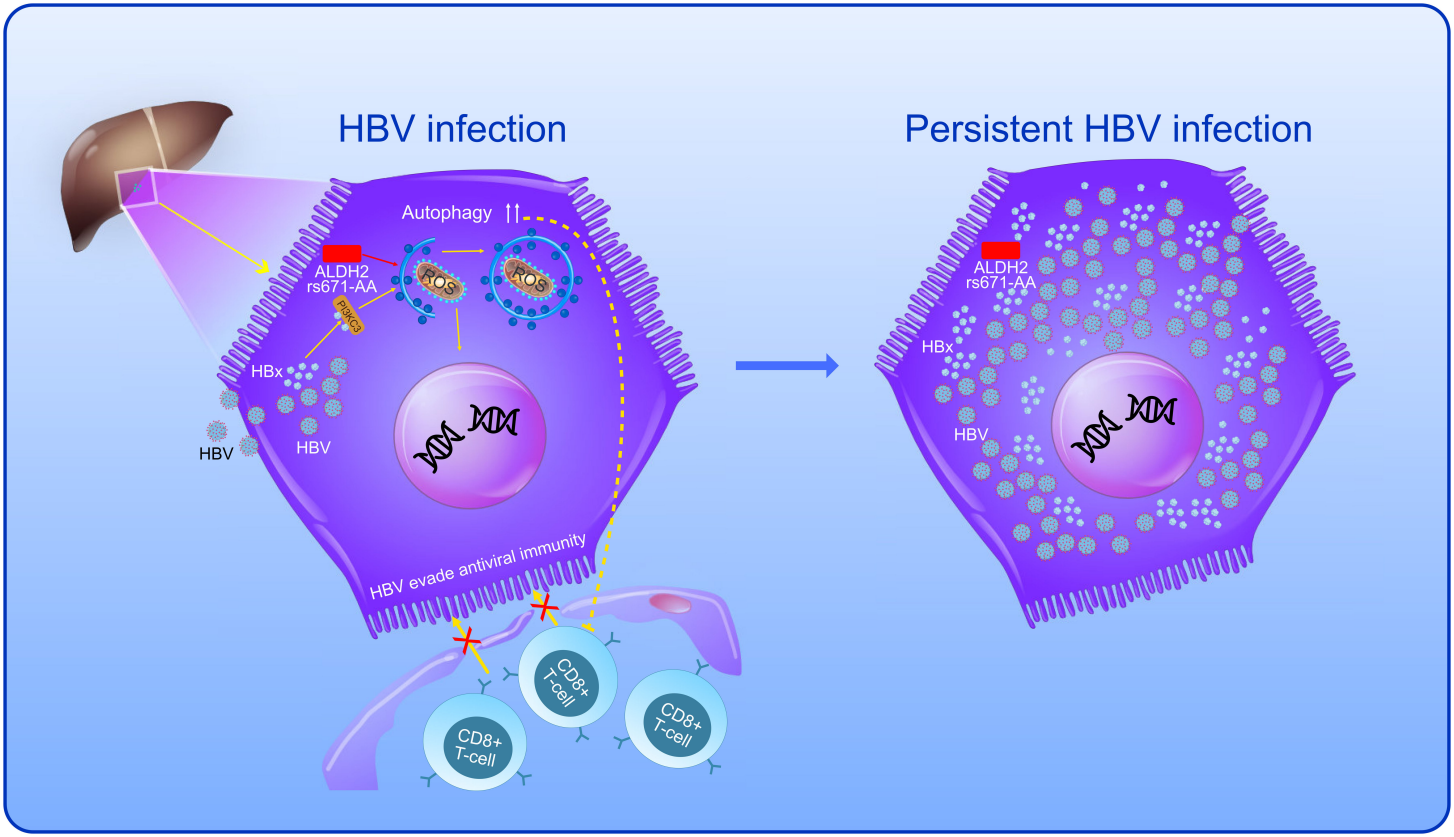
The possible hazardous role of the rs671-AA variant during HBV infection and persistence. The autophagic pathway in HBV-infected hepatocytes is enhanced by hepatitis B virus × protein (HBx) *via* the binding to phosphatidylinositol 3-kinase class III (PI3KC3). Meanwhile, ALDH2-rs671-AA mutant in individuals with decreased activity of ALDH2 enzymes might also activate the autophagy signal pathway, triggering autophagy then promoting HBV to evade antiviral immunity, permitting the survival of virus-infected hepatocytes, further enhancing the HBV replication and ultimately promoting the persistence of HBV infection.

In the present study, individuals with a high HBV-DNA level accounted for a larger proportion of patients with CHB with the rs671-AA genotype compared to the rs671-GG/GA genotype, indicating a significant positive association between HBV-DNA level and the ALDH2 rs671-AA genotype. We reasonably conclude that the decreased activity of the ALDH2 enzymes in patients with CHB, which resulted from the rs671-AA mutant (24), might activate the autophagy signal pathway (10), triggering autophagy then promoting HBV to evade antiviral immunity (29), permitting the survival of virus-infected cells (29), further enhancing the HBV replication (5, 6), and ultimately promoting the persistence of HBV infection (8). The present study demonstrates the possible hazardous role of the rs671-AA variant during HBV infection and persistence (Figure 3).

However, the decreased activity of ALDH2 resulting from the rs671-AA mutant could trigger contrary effects in the incidence of HCC. There were lower distribution frequencies of the rs671-AA genotype in the HCC group compared with the CHB group in subgroup analysis, which was in accordance with the lower HBV-DNA quantitation in the patients with HCC. Combined with the lower HBV-DNA level in the HCC group, we speculate that the rs671-AA mutant might be a potential risk predictor of HCC incidence in the CHB group. Thus, CHB individuals with ALDH rs671-AA genotype potentially have a lower risk of incidence of HCC. Recently, Seo et al. (31) have reported that the progression of HCC was mainly observed in patients with the ALDH2-rs671-GG genotype rather than the GA/AA genotype (31).

There are some limitations to our study. First, the selected hot spots (rs671 and rs1229984) might miss other important mutant sites, including sites that may be in linkage disequilibrium with the selected sites. It would be better to sequence the whole genome of ADH1B and ALDH2 to discover new loci that might play significant roles in the pathogenesis of CHB or other HBV-related liver diseases. Second, we did not assess the influence of ALDH2 expression mediated by rs671 mutants in liver tissues due to the limited acquisition of liver biopsies in these patients. Third, there were only 97 patients with HCC enrolled in the present study, the statistical conclusion that subjects with rs671-AA genotype might have a lower risk of HCC incidence needs a larger sample size to confirm this conclusion. Finally, we did not verify the underlying mechanism of ALDH2 rs671 mutant *in vivo* or *in vitro*, it would be performed in our future studies.

In conclusion, the present study provides evidence for the perspective that the ALDH2-rs671 variant was correlated with HBV infection and persistence. It is the first time that the positive association between rs671 polymorphism and HBV infection has been investigated. Currently, the hot site rs671-AA imparts a hazardous role during persistent HBV infection. These results might shed light on the study of HBV susceptibility of individuals and the prevention of persistent HBV infection, and the targeting of drugs for a functional cure of patients with CHB.

## Data Availability Statement

The datasets presented in this study can be found in online repositories. The names of the repository/repositories and accession number(s) can be found in the article/Supplementary Material.

## Ethics Statement

The studies involving human participants were reviewed and approved by the Ethics Committee of Shanghai Ruijin Hospital, School of Medicine, Shanghai Jiaotong University. The patients/participants provided their written informed consent to participate in this study.

## Author Contributions

QX, XX, and GZ conceived, designed, and directed the overall project, reviewed the manuscript, and approved the final version. HW and WC directed partial experiments. PW, GZ, WT, and DS collected samples. RL, ZL, and XW collected data. PW and RM performed experiments and analyzed the data. PW and XX wrote the manuscript. All the authors had access to the study data and have reviewed and approved the final manuscript.

## Funding

This study was supported by the National Natural Science Foundation of China (Nos. 82170619, 81970544, 82070604, 81770587, 81770578, and 81900527), the Three-Year Public Health Action Plan (2020–2022) of Shanghai (No. GWV-10.1-XK13), the Shanghai Municipal Key Clinical Specialty (shslczdzk01103), the Shanghai Ruijin Hospital Clinical Skills and Innovations (2018CR005), the Shanghai talent development fund (2020097), the Shanghai Rising Stars of Medical Talent Youth Development Program Outstanding Youth Medical Talents [SHWJRS(2021)-99], the Shanghai Outstanding Academic Leader Youth Program (20XD1422600) and the Shanghai Sailing Program (No. 19YF1429200).

## Conflict of Interest

The authors declare that the research was conducted in the absence of any commercial or financial relationships that could be construed as a potential conflict of interest.

## Publisher's Note

All claims expressed in this article are solely those of the authors and do not necessarily represent those of their affiliated organizations, or those of the publisher, the editors and the reviewers. Any product that may be evaluated in this article, or claim that may be made by its manufacturer, is not guaranteed or endorsed by the publisher.
