# Supplementary material for: Genetic Variations of ALDH (rs671) Are Associated With the Persistence of HBV Infection Among the Chinese Han Population
Source: Front Med (Lausanne). 2022 Feb 14;9:811639. doi: 10.3389/fmed.2022.811639 (PMC8882735; doi:10.3389/fmed.2022.811639)
Supplement: Supplementary file 1 [file Data_Sheet_1.docx]

# Genetic Variations of ALDH (rs671) is associated with the Persistent

# Hepatitis B Virus Infection among Han Chinese

Authors:

Dabao Shang^1,2,^ ^†^, Peng Wang^1,3, †^, Weiliang Tang^1,2, †^, Ruidong Mo^1,2^, Rongtao Lai^1,2^, Jie Lu^1,2^, Ziqiang Li^1,2^, Xiaolin Wang^1,2^, Wei Cai^1,2^, Hui Wang^1,2^, Gangde Zhao^1,2,^* , Qing Xie^1,2,^* , Xiaogang Xiang^1,2,^*

^1^Department of Infectious Diseases, Ruijin Hospital, Shanghai Jiaotong University Medicine, China

^2.^Translational Lab of Liver Diseases, Department of Infectious Diseases, Ruijin Hospital, Jiaotong University School of Medicine, China

^3^ Department of Hepatobiliary Medicine, Eastern Hepatobiliary Surgery Hospital, Naval Medical University, Shanghai 200438, China.

^†^These authors have contributed equally to this work

*Correspondence author: Dr. Xiaogang Xiang or Dr. Qing Xie or Dr. Gangde Zhao

Department of Infectious Diseases, Ruijin Hospital, Shanghai Jiaotong University School of Medicine, Shanghai 200025, China

Tel: 86-21-64370045-680403

Fax: 86-21-64454930

Email: shine-xxg@163.com or xieqingrjh@163.com or zhaogd1016@foxmail.com

**Supplementary Table 1. PCR primers sequence for ADH1B and ALDH2 genotyping.**

| Rs ID | SNP Property | Base Pair Change | Amino acid Chang |  | PCR Primers |
| --- | --- | --- | --- | --- | --- |
| rs1229984 | extron | G>A | P.His48Arg |  | F: CTGAATCTGAACAGCTTCTC |
|  |  |  |  |  | R: TTGCCACTAACCACGTGGTC |
|  |  |  |  |  | S: TTGGTGGCTGTAGGAATCTGTC |
| rs671 | extron | G>A | p.Glu457Lys |  | F: TTGGTGGCTACAAGATGTCG |
|  |  |  |  |  | R: AGGTCCCACACTCACAGTTT |
|  |  |  |  |  | S: TTTCCACACTCACAGTTTTCACTT |

**Supplementary Table 2. The call rate, Hardy-Weinberg Equilibrium test and frequencies for the 6 SNPs in the study.**

| Rs ID |  | Chr | Position | MA | Call rate (%) | | | HWE* | | Freq(A1) | |
| --- | --- | --- | --- | --- | --- | --- | --- | --- | --- | --- | --- |
|  |  |  |  |  | Controls | Patients | overall | Controls | Patients | Controls | Patients |
| rs671 | Hepatitis B | 12 | 74568710 | A | 100 | 100 | 100 | 0.125 | 0.34 | 24.11 | 26.72 |
|  | AHB |  |  |  | 100 | 100 | 100 | 0.125 | 1 | 24.11 | 22.22 |
|  | CHB |  |  |  | 100 | 100 | 100 | 0.125 | 0.395 | 24.11 | 28.79 |
|  | ACLF |  |  |  | 100 | 100 | 100 | 0.125 | 0.161 | 24.11 | 27.53 |
|  | LC |  |  |  | 100 | 100 | 100 | 0.125 | 0.524 | 24.11 | 24.53 |
|  | HCC |  |  |  | 100 | 100 | 100 | 0.125 | 1 | 24.11 | 22.16 |
| rs1229984 | CHB | 4 | 40396781 | G | 100 | 100 | 100 | 1 | 0.777 | 28.74 | 28.76 |

Chr: Chromosome; MA: Minor Allele; * Hardy-Weinberg Equilibrium test; Freq: Frequency of A1.

**Supplementary Table 3 Demographic and clinical features of the patients and healthy controls in the study of the combination role of ADH1B and ALDH2 associated with the CHB risk.**

| **Characteristic** | **HC** (n=287) | **CHB** (n=266) | OR(95%CI) | ***P*-value** |
| --- | --- | --- | --- | --- |
| **Mean Age**† | 41.61±12.79 | 43.13±14.01 | / | 0.435 |
| **Gender** (no, %）†† | 147(51.22) | 144(54.14) | / | 0.459 |
| **Tbil** (umol/L) † | 15.23±4.28 | 98.20±160.1 | / | <0.0001 |
| **ALT** (IU/mL) † | 22.00±9.12 | 296.5±454.2 | / | <0.0001 |
| **AST** ( IU/mL) † | 21.12±4.64 | 176.6±248.7 | / | < 0.0001 |
| **GGT**( IU/mL) † | 18.49±11.10 | 77.27±86.53 | / | < 0.0001 |
| **AFP** (ug/L) † | 1.98±1.69 | 209.9±106.1 | / | 0.0015 |
| **eAg +** (no,%) †† | / | 143(53.76) | / | / |
| **HBV-DNA** [Log_10_(copies)/mL] † |  | 5.828±1.614 | / | / |
| <10^3^†† | / | 60(22.56) | / | / |
| 10^3^-10^5^†† | / | 62(23.31) | / | / |
| >10^5^†† | / | 144(54.13) | / | / |
| **rs1229984**† |  |  |  |  |
| **A** | 409(71.26) | 379(71.24) | 1 | / |
| **G** | 165(28.74) | 153(28.76) | 1.001(0.771-1.299) | 0.996 |
| AA | 150(52.26) | 141(53.0) | 1 | / |
| GA | 109(37.98) | 97(36.47) | 0.947(0.662-1.353) | 0.785 |
| GG | 28(9.76) | 28(10.53) | 1.124(0.622-2.029) | 0.764 |
| **rs671**† |  |  |  |  |
| **G** | 425(74.04) | 387(72.74) | 1 |  |
| **A** | 149(25.96) | 145(27.26) | 1.069(0.818-1.396) | 0.634 |
| GG | 152(52.96) | 129(48.49) | 1 | / |
| GA | 121(42.16) | 129(48.49) | 1.256(0.893-1.768) | 0.193 |
| AA | 14(4.88) | 8(3.02) | 0.536(0.217-1.323) | 0.189 |

HC: Health control; CHB: Chronic Hepatitis B; †Date presented as (Mean ± SD); †† Date presented as (n, %); *P*: *t* test for all of CH HC individuals.
